# Retraction Note: The PI3K/mTOR Dual Inhibitor BEZ235 Nanoparticles Improve Radiosensitization of Hepatoma Cells Through Apoptosis and Regulation DNA Repair Pathway

**DOI:** 10.1186/s11671-021-03638-4

**Published:** 2021-12-16

**Authors:** Xiaolong Tang, Amin Li, Chunmei Xie, Yinci Zhang, Xueke Liu, Yinghai Xie, Binquan Wu, Shuping Zhou, Xudong Huang, Yongfang Ma, Weiya Cao, Ruyue Xu, Jing Shen, Zhen Huo, Shuyu Cai, Yong Liang, Dong Ma

**Affiliations:** 1 Huainan First People’s Hospital and First Affiliated Hospital of Medical School, Anhui University of Science and Technology, Huainan, 232001 People’s Republic of China; 2 Blood Transfusion Department, Guangzhou 8th People’s Hospital, Guangzhou Medical University, Guangzhou, 510632 People’s Republic of China; 3 Department of Hepatobiliary Surgery, The First Affiliated Hospital of Bengbu Medical College, Bengbu, 233004 People’s Republic of China; 4 Department of Interventional, Affiliated Oriental Hospital, Anhui University of Technology, Huainan, 232003 People’s Republic of China; 5 Huai’an Hospital Affiliated of Xuzhou Medical College and Huai’an Second Hospital, Huai’an, 223002 People’s Republic of China; 6 Key Laboratory of Biomaterials of Guangdong Higher Education Institutes, Department of Biomedical Engineering, Jinan University, Guangzhou, 510632 People’s Republic of China

## Retraction Note: Tang et al. Nanoscale Research Letters (2020) 15:63 https://doi.org/10.1186/s11671-020-3289-z

The Editors-in-Chief have retracted this article because there appear to be anomalies in four of the figures, specifically Figure 5, Figure 6A, Figure 6C and Figure 8A. The Editors-in-Chief therefore no longer have confidence in the data presented. The authors have been invited to submit a new manuscript for peer review. Xialong Tang stated on behalf of all co-authors that they do not agree with this retraction.

